# Uniting for Ukraine Tuberculosis Screening Experience, San Francisco, California, USA

**DOI:** 10.3201/eid2908.230347

**Published:** 2023-08

**Authors:** Janice K. Louie, Rocio Agraz-Lara, Laura Romo, Cristy Deiterich, Cathleen Xing, Susannah Graves

**Affiliations:** University of California, San Francisco, California, USA (J.K. Louie);; San Francisco Department of Public Health, San Francisco (J.K. Louie, R. Agraz-Lara, L. Romo, C. Xing, S. Graves);; San Francisco Department of Public Health Community Health Equity and Promotion Branch’s Refugee Health Assessment Program, San Francisco (C. Deiterich)

**Keywords:** tuberculosis, TB, tuberculosis and other mycobacteria, bacteria, Ukraine, parolees, screening, interferon-γ-release assay, IGRA, Uniting for Ukraine Tuberculosis Screening Experience, U4U, San Francisco, California, United States

## Abstract

Ukraine surveillance data suggest high tuberculosis (TB) incidence, including multidrug resistance. Of 299 newcomers from Ukraine screened in San Francisco, California, USA, by using an interferon-γ-release-assay (IGRA) and chest radiograph, 7.4% were IGRA positive and 1 had laboratory-confirmed pansusceptible TB. Screening with IGRA and chest radiograph can help characterize TB risk.

World Health Organization surveillance data estimate that Ukraine has the fourth highest tuberculosis (TB) incidence in the European Region, at 71 cases/100,000 population in 2021 (*1**–**3*). Ukraine is believed to have a high burden of rifampin- and multidrug-resistant TB, accounting for ≈31% of culture-confirmed cases in 2021 (*2*,*3*). In addition, 22% of persons from Ukraine who have TB are infected with HIV; TB is the leading cause of death in this population (*2*,*3*).

In April 2022, the US Department of Homeland Security announced the Uniting for Ukraine (U4U) program to provide a pathway for citizens from Ukraine to enter the United States under humanitarian parole (*4*). U4U requires that parolees >2 years of age submit an attestation to the US Citizenship and Immigration Services confirming that TB screening with symptom review and an interferon-γ-release assay (IGRA) are performed within 90 days of US entry (*5*). In response, the San Francisco Department of Public Health TB Clinic partnered with the SFDPH Newcomers Health Program, a county Refugee Health Assessment Program, to reach out to community, professional, and faith-based groups to encourage expedited, no-cost TB screening to promptly identify and treat any U4U parolees who had latent or active TB (*6*,*7*).

## The Study

To meet attestation requirements, U4U parolees who had a San Francisco address were screened for TB symptoms (fever, cough, night sweats, weight loss, fatigue, or hemoptysis) and tested by using the IGRA QuantiFERONTB Gold In-Tube Test (https://www.quantiferon.com). HIV testing was offered to all persons >2 years of age, and a chest radiograph was offered to persons >15 years of age to aid early identification of pulmonary TB. Parolees who had a positive IGRA result but unremarkable chest radiograph were offered latent TB infection (LTBI) treatment according to US Centers for Disease Control and Prevention recommendations (*8*).

Patients who had chest radiograph abnormalities suggestive of TB, regardless of IGRA result, underwent further evaluation by collection of 3 sputum samples for acid-fast bacilli (AFB) smear and culture and 1 sputum sample for nucleic acid amplification testing (NAAT) with the GeneXpert MTB/RIF assay (Cepheid, https://www.cepheid.com), which tests for TB and rifampin resistance. If sputum cultures were negative, LTBI treatment was recommended (*8*). Patients who had positive results for TB by NAAT or AFB culture were given treatment for active TB according to national recommendations (*9*).

During May 10, 2022‒April 14, 2023, a total of 299 U4U parolees underwent TB screening (Table). Median age was 33 years (range 8 months‒84 years); 116 (38.8%) were male. All patients denied previous active or latent TB. Of 298 patients >2 years of age, 274 (91.9%) agreed to HIV testing; all showed negative results. None of the 299 patients screened reported alcohol or substance use or previous incarceration. Three (1.0%) patients reported having a medical TB risk factor; all 3 had diabetes. All patients denied TB symptoms except for 1 (described later in this report). 

**Table:** Characteristics of 299 parolees from Ukraine screened for tuberculosis, San Francisco, California, USA, May 2022‒April 2023*

Characteristic	Value
Sex	
F	183 (61.2)
M	116 (38.8)
Median age, y (range)	33 (0.75–84)
TB medical risk factor per patient report	
Diabetes mellitus	3/299 (1.0)
HIV infection	0/299 (0.0)
Alcohol or substance use	0/299 (0.0)
Other†	0/299 (0.0)
Positive HIV test result‡	0/274 (0.0)
Positive IGRA result	22/299 (7.4)
Median age, y (range) with positive IGRA result	51.5 (17–81)
Chest radiograph performed§	240/245 (98.0)
Abnormalities on chest radiograph, all	7/240 (2.9)
Median age, y (range) with abnormal chest radiograph	43 (36–63)
Abnormalities on chest radiograph, IGRA negative	4/240 (1.7)
Diagnosis of active TB	1/299 (0.33)
LTBI treatment recommended	
Received treatment for LTBI/IGRA positive result	14/22 (63.6)
Not treated for LTBI/IGRA positive result	8/22 (36.4)
Refused treatment	5/22 (22.7)
Pending start of LTBI treatment	3/22 (13.6)

*Values are no. (%) or no. positive/no. tested (%) except as indicated. IGRA, interferon-γ-release assay; LTBI, latent TB infection; TB, tuberculosis**.**†Other medical TB risk factors: severe kidney disease, silicosis, low bodyweight, organ transplant, head and neck cancer, and treatment with immunosuppressive agents (*10*). ‡All 298 parolees >2 years of age were offered HIV testing; 274 (91.9%) agreed. §All 245 parolees ≥15 y of age were offered chest radiograph screening; 240 (98.0%) agreed.

Of the 299 patients, 22 (7.4%) had positive IGRA results; median age was 51.5 (range 17–81) years. Of 245 patients >15 years of age, 240 (98.0%) received a chest radiograph. Seven (2.9%) patients had abnormal chest radiograph results, consistent with possible TB, including 4 patients who had negative IGRA results; median age was 43 (range 36–63) years.

One parolee had laboratory confirmation of active TB. The patient reported productive cough and rhinorrhea for 10 days but no other TB symptoms. The patient had no epidemiologic or medical risk factors; HIV test result was negative, IGRA test result was positive, and chest radiograph identified upper lobe nodules. Sputum samples tested showed few AFB smear-positive, NAAT-positive results without rifampin resistance and grew *Mycobacterium tuberculosis* that was pansusceptible to isoniazid, rifampin, ethambutol, and pyrazinamide. The patient received TB therapy; all household contacts, including a child <5 years of age, tested negative by IGRA at baseline and 8–10 weeks later.

## Conclusions

Despite surveillance data reporting high TB incidence (including drug-resistant TB) in Ukraine, only 7.4% of parolees in this investigation received diagnoses of LTBI, and only 1 had laboratory-confirmed, pansusceptible, active pulmonary disease (*1**‒**3*). Most parolees were female, possibly reflecting that many men have remained in Ukraine during wartime. All parolees with LTBI were >18 years of age, consistent with reports that TB is uncommon in children from Ukraine (*1**‒**3*). Most parolees reported no concurrent medical condition, and none tested were HIV positive. The percentages of U4U parolees testing positive by IGRA was low compared with other San Francisco immigrant populations; in the past 5 years, of clients undergoing screening for homeless shelter housing, 20.2% who originated from Mexico and 27.5% from Central America (including Belize, El Salvador, Guatemala, Honduras, and Nicaragua) have tested IGRA positive (San Francisco Department of Public Health, unpub. data).

U4U parolees might not be representative of populations from Ukraine most likely to be given a diagnosis of TB. For example, data for Ukraine for 2021 suggest that HIV, alcohol use, malnutrition, and diabetes are major TB risk factors; those factors were uncommon or absent in the San Francisco U4U population (*1**‒**3*). Our numbers are reflective of the U4U program only and might not be generalizable to other jurisdictions. Nevertheless, vigilance in the U4U population remains warranted because armed conflict and mass displacements have historically been associated with increases in TB incidence, drug-resistant TB, and TB deaths, possibly caused by disruptions in healthcare services, malnutrition, and need for temporary housing with associated crowding and poor hygiene (*11*).

The Centers for Disease Control and Prevention Division of Global Migration and Quarantine has established requirements for overseas screening of new refugees before entry into the United States, which include medical history, physical examination, and TB screening (*12*). For persons originating from countries that have a TB incidence of >20 cases/100,000 persons, the overseas screening requirement for persons >15 years of age includes a chest radiograph (IGRA optional); for those 2–14 years of age, only IGRA is necessary (*12*). Within 90 days of US arrival, a domestic screening, including history, physical examination, and review of overseas screening results, is recommended; depending on the person, refugees might undergo further evaluation for LTBI (if overseas IGRA was not performed or the result is >6 months old) or active TB (if new symptoms or physical examination abnormalities have developed since overseas screening) (*13*).

In humanitarian situations through which newcomers enter the United States emergently from high-incidence countries without previous overseas evaluation, domestic TB screening with IGRA and chest radiograph (in persons >15 years of age) might be merited to match existing overseas refugee screening recommendations. Because IGRA can show false-negative results for >20% of persons who have active TB, addition of a chest radiograph can help enable rapid and sensitive detection of pulmonary TB, ensure prompt treatment, and prevent local transmission (*14*). Our inclusion of chest radiographs also provides reassuring data suggesting that infectious pulmonary TB is not being missed in U4U parolees entering San Francisco, despite the high incidence reported in surveillance data for Ukraine.

In late 2022 and early 2023, the Department of Homeland Security implemented programs similar to U4U for new parolees from Venezuela, Nicaragua, Cuba, and Haiti (*15*). Those parolees have not been screened overseas, have the same TB attestation requirements as U4U, and might have entered the United States under circumstances that convey higher TB risk (e.g., extreme poverty, expolitical prisoners, or long and crowded land journeys) (*15*). The establishment of U4U screening has enabled SFDPH to assess those populations similarly. To date, of 38 parolees from Venezuela and Nicaragua screened, LTBI has been identified in 3 from Nicaragua; an additional asymptomatic parolee from Nicaragua with a negative IGRA result was given a diagnosis of smear-positive active pulmonary TB. Parolee screening by both IGRA and chest radiograph has provided SFDPH with timely and informative data (including positive and negative results) about TB risk in the diverse parolee populations from high-incidence countries.
